# Evaluation of the diagnostic potential of urinary *N*-Acetyltyramine-*O*,β-glucuronide (NATOG) as diagnostic biomarker for *Onchocerca volvulus* infection

**DOI:** 10.1186/s13071-016-1582-6

**Published:** 2016-05-23

**Authors:** Ole Lagatie, Emmanuel Njumbe Ediage, Linda Batsa Debrah, Luc Diels, Christ Nolten, Petra Vinken, Alex Debrah, Lieve Dillen, Steven Silber, Lieven J. Stuyver

**Affiliations:** Janssen Diagnostics, Janssen R&D, Turnhoutseweg 30, 2340 Beerse, Belgium; Bioanalysis, Janssen R&D, Turnhoutseweg 30, 2340 Beerse, Belgium; Kumasi Centre for Collaborative Research, Kwame Nkrumah University of Science and Technology, Kumasi, Ghana; Neuroscience, Janssen R&D, Turnhoutseweg 30, 2340 Beerse, Belgium; Toxicology, Janssen R&D, Turnhoutseweg 30, 2340 Beerse, Belgium; Faculty of Allied Health Sciences, Kwame Nkrumah University of Science and Technology, Kumasi, Ghana; Janssen Global Public Health, Janssen R&D, 12 W Sunset Ave, Philadelphia, PA 19118 USA

**Keywords:** *Onchocerca volvulus*, River blindness, Onchocerciasis, Biomarker, NATOG, Urine, Diagnostic

## Abstract

**Background:**

Onchocerciasis, also known as river blindness is one of the neglected tropical diseases affecting millions of people, mainly in sub-Saharan Africa and is caused by the filarial nematode *Onchocerca volvulus*. Efforts to eliminate this disease are ongoing and are based on mass drug administration programs with the microfilaricide ivermectin. In order to monitor the efficacy of these programs, there is an unmet need for diagnostic tools capable of identifying infected patients. We have investigated the diagnostic potential of urinary *N*-acetyltyramine-*O*,β-glucuronide (NATOG), which is a promising *O. volvulus* specific biomarker previously identified by urine metabolome analysis.

**Methods:**

A liquid chromatography tandem mass spectrometry (LC-MS/MS) method was used to assess the stability characteristics of NATOG and to evaluate the levels of NATOG in study samples. An LC-fluorescence method was also developed.

**Results:**

Stability characteristics of NATOG were investigated and shown to be ideally suited for use in tropical settings. Also, an easy and more accessible method based on liquid chromatography coupled to fluorescence detection was developed and shown to have the necessary sensitivity (limit of quantification 1 μM). Furthermore, we have evaluated the levels of NATOG in a population of 98 nodule-positive individuals from Ghana with no or low levels of microfilaria in the skin and compared them with the levels observed in different control groups (endemic controls (*n* = 50), non-endemic controls (*n* = 18) and lymphatic filariasis (*n* = 51). Only a few (5 %) of nodule-positive individuals showed an increased level (> 10 μM) of NATOG and there was no statistical difference between the nodule-positive individuals and the control groups (*P* > 0.05).

**Conclusions:**

Results of the present study indicate the limited potential of NATOG as a diagnostic biomarker for *O. volvulus* infection in amicrofilaridermic individuals.

**Electronic supplementary material:**

The online version of this article (doi:10.1186/s13071-016-1582-6) contains supplementary material, which is available to authorized users.

## Background

Onchocerciasis, commonly known as river blindness is a neglected tropical disease caused by infection with the filarial nematode, *O. volvulus*. More than 99 % of infected people live in Africa and at least 120 million people living in these endemic areas are at risk of infection [1, 2]. The last comprehensive survey conducted in 2008 indicated that 26 million people were infected with onchocerciasis, of whom 265,000 were blind and 746,000 were visually impaired. In addition, approximately 4 million people suffer from onchodermatitis with severe itching [3]. Since the introduction of Ivermectin and the initiation of the mass drug administration programs (MDA) in 1987, hundreds of millions of people have been treated with a resultant reduction in both visual impairment and symptomatic onchodermatitis.

Diagnostic tools for detection of *O. volvulus* infection traditionally were limited to detection of microfilaria (mf) in small, superficial skin biopsy samples (skin snips) [4]. More recently rapid-format tests for the detection of IgG4 antibodies to the parasitic antigen Ov-16 were developed [5–10]. Serological antibody tests using recombinant antigens however cannot distinguish between past and active infection. Another shortcoming is their inability to monitor disease progression or monitor treatment response. Several efforts have therefore been undertaken to identify novel biomarkers that do not suffer from these shortcomings [11]. This has already successfully been done for *Dirofilaria immitis* (heartworm) and *Wuchereria bancrofti* (the most prevalent cause of lymphatic filariasis), for which sensitive assays have been developed that detect circulating filarial antigens in the blood [12–15]. Also circulating parasitic microRNAs have been suggested as potential biomarkers for filarial infection, but it is not clear yet whether this will provide the required specificity, sensitivity and dynamic range (for treatment monitoring and determination of cure) to be useful as diagnostic biomarker for *O. volvulus* infection [16–23].

One approach that has shown promise is the use of metabolome analysis of serum samples from infected individuals, but the complexity of the identified set of biomarkers hampered its further development [24]. Subsequent work using similar technology, but on urine samples, has identified urinary *N*-acetyltyramine-*O*,β-glucuronide (NATOG) as a unique biomarker for *O. volvulus* infection [25]. This molecule is a neurotransmitter-derived secretion molecule from *O. volvulus*.

In the work presented here we have further investigated the potential of NATOG as a diagnostic marker by evaluating several features. A liquid chromatography tandem mass spectrometry (LC-MS/MS) method was optimized and used for all preliminary investigations and also for the analysis of study samples. First, its stability under conditions resembling real-life conditions in a tropical environment as well as adsorption characteristics was investigated. We have also investigated the use of LC-fluorescence for detection of NATOG in urine, which would open the path for development of a microfluidic point-of-care device. Furthermore, NATOG levels in urine samples collected from nodule-positive individuals in Ghana, but with no or very low levels of microfilaria in the skin, were assessed and compared to several control groups in order to determine the diagnostic performance of this biomarker.

## Methods

### NATOG reference material

*N*-acetyltyramine-*O*,β-glucuronide (NATOG) was synthesized by WuXi AppTec Co., Ltd (Hong Kong, China). Purity and structure were determined by LC-MS, ^1^H-NMR and SFC and confirmed to be > 98 % NATOG. Product was dissolved in Milli-Q water for further use.

### Collection of human urine samples

Urine samples were collected as part of a field study in Ghana. This study was undertaken in an onchocerciasis-endemic community located in Adansi South District along the Pra river basins in the Ashanti Region of Ghana. This study was approved by the Committee on Human Research, Publications and Ethics of the School of Medical Sciences of the Kwame Nkrumah University of Science and Technology, Kumasi, Ghana and study subjects signed an informed consent form. Physical examinations were performed to identify those subjects having palpable nodules. Skin snips (biopsies) were then taken from those with nodules in order to determine the microfilarial (mf) load in the skin [26]. Most subjects were participating in mass drug administration programs. A total of 98 nodule-positive subjects that donated urine samples were included, as well as 50 endemic controls that had no visible signs of onchocerciasis. Additionally, urine samples from 18 non-endemic controls (from Kumasi, Ashanti Region) and 51 lymphatic filariasis patients (from Ahanta West District, Western Region) were available for testing. An overview of the patient demographics is provided in Table 1.

**Table 1:** Demographic information of study population investigated

Characteristic	Group			
	Nodule positive	Endemic controls	Non-endemic controls	Lymphatic filariasis
No. of subjects	98	50	18	51
Age, median (Min-Max)	47 (21–85)	35 (18–81)	25 (13–52)	33 (18–68)
Gender, *n* (%)				
Male	53 (53)	25 (50)	12 (67)	37 (73)
Female	45 (47)	25 (40)	6 (33)	14 (27)
No. of nodules, median (Min-Max)	1 (1–5)	0	na	na
mf status, *n* (%)				
0 mf/mg	87 (89)	50 (100)	na	na
0–5 mf/mg	10 (9)	0 (0)	na	na
5–10 mf/mg	1 (1)	0 (0)	na	na
No. of IVM rounds, median (Min-Max)	2 (0–10)	0 (0–1)	na	na
Time since last ivermectin treatment, *n* (%)				na
Not treated	17 (17)	33 (66)	na	na
< 20 months	67 (68)	5 (10)	na	na
> 20 months	14 (14)	12 (24)	na	na
Ov16 status, *n* (%)				
Positive	68 (69)	25 (50)	0 (0)	12 (24)^a^
Negative	30 (31)	25 (50)	18 (100)	38 (75)

^a^For one LF subject, no Ov16 status has been determined

### Sample preparation

For preparation of calibration samples, Quality Control (QC) samples and stability samples, urine samples were obtained from normal healthy subjects in Belgium. Samples were blinded and no link to the different donors was maintained. All calibration, QC and stability samples were prepared by spiking aqueous stock solutions of NATOG to Milli-Q water or human urine, as indicated. For investigation of spike recovery of NATOG using the LC-Fluorescence assay, urine samples collected from healthy subjects were used [27–31]. Before analysis, study samples were diluted 30-fold in Milli-Q water for LC-MS/MS analysis or 10-fold for LC-Fluorescence analysis.

### LC-MS/MS Analysis

The samples (2-μl injection volume) were chromatographically separated using an UPLC (Acquity UPLC; Waters Corporation, Etten-Leur, Nederland) system on Acquity HSS T3 column (1.8 μm, 2.1 mm × 50 mm; Waters Corporation) and analyzed with a Triple quadrupole MS/MS (API 4000, AB Sciex). The column temperature was maintained at 45 °C. Eluting buffers were buffer A (0.1 % HCOOH in H_2_O) and buffer B (MeOH). Starting conditions were 2 % buffer B at a flow rate of 0.6 ml/min. Over 3 min a gradient was applied to 15 % B. Thereafter the percentage of buffer B was increased to 98 % in a step gradient, followed by an isocratic hold for 0.9 min before returning to the starting conditions. Data were collected using SRM in positive ESI mode with Q1 Mass 356.1 Da and Q3 Mass 180.1 Da at a dwell time of 100 ms. The capillary voltage was 2,500 V; the nebulizer pressure, drying gas flow and gas temperature were set to 20 psig, 7 l/min, and 500 °C, respectively. A linear regression curve was calculated after log-log transformation, based on the data of the calibration samples and used for determination of NATOG concentration in unknown samples. Calculations were performed using Analyst 1.6.2 (Sciex).

### LC-Fluorescence Analysis

Analysis was similar to the LC-MS/MS analysis except for the detector type which in this case was a Waters 474 fluorescence detector (Waters Corporation). The gradient was slightly adapted to separate co-eluting peaks which would otherwise result in quenching of the NATOG signal: starting conditions were 10 % buffer B at a flow rate of 0.6 ml/min. After 3 min the percentage of buffer B was increased to 98 % in a step gradient, followed by an isocratic hold for 0.9 min before returning to the starting conditions. The fluorescence detection wavelengths were set at λex/λem = 232/290. A linear regression curve was calculated based on the data of the calibration samples and used for determination of NATOG concentration in unknown samples. Calculations were performed using Empower 3 (Waters Corporation).

### Acquisition of 2D fluorescence spectra

The 2D fluorescence spectra of NATOG solutions, urine and milli-Q water were obtained with a Tecan Infinite M1000 fluorimeter. The excitation source was a Xenon Flash lamp, and the detector was a gated photomultiplier. Double monochromators with selectable bandwidth were used on both excitation and emission sides. Excitation and emission slits were 2.5 and 5 nm, respectively. Fluorescence spectra were generated in the range 230 to 300 nm (excitation) and 280 to 350 nm (emission), with an excitation and emission wavelength incrementing step of 2 nm.

### Adsorption, temperature and light stability evaluation of NATOG

The stability of NATOG in different pH (pH 4, 6 and 8) and temperature conditions (4 °C, room temperature and 50 °C) for a 24 h period were evaluated with spiked blank urine samples (spiked with 5.63 μM NATOG). Possible adsorption onto glass and polypropylene recipients was evaluated using the same spiked urine samples. Urine was spiked (in replicate), vortexed and the content was completely poured into another tube. This cycle was repeated 5 more times (six tubes in total). The concentration in tube 1 and 6 were used to determine the amount adsorbed. For the light (in)stability test, a suntester (SUNTEST CPS+, Atlas MTS) was used. Samples were incubated in clear and amber glass recipients for 0.5 and 3 h at 500 W light intensity. Incubation in the suntester for 5 min corresponds to 1 h direct sunlight exposure.

### Urinary creatinine measurement

Urinary creatinine was measured by an enzymatic method on a Cobas® 6000 c 501 clinical chemistry analyzer (Roche Diagnostics, Mannheim, Germany) using Roche reagents.

### Statistical analysis

For comparison of different groups, one-tailed Mann-Whitney *U* test and ROC analysis were performed using GraphPad Prism version 6.02. *P*-value < 0.05 was considered to be significantly different.

## Results

### Performance characteristics of LC-MS/MS assay for NATOG quantification in urine

**Fig. 1:**
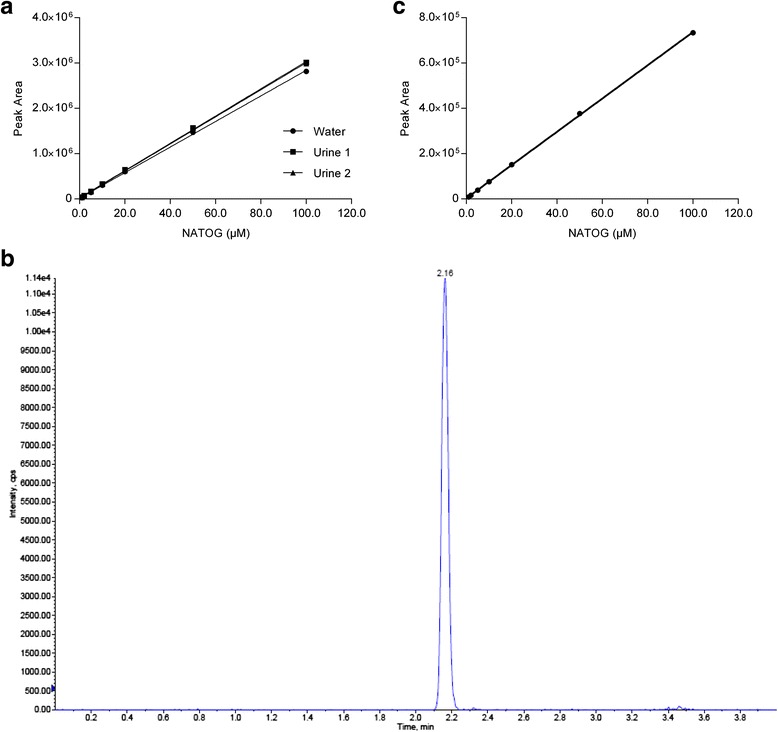
**a** UPLC-MS/MS Calibration curves of NATOG prepared in water and urine. **b** UPLC-MS/MS chromatogram of a urine sample with an endogenous NATOG concentration of 0.31 μM. **c** LC-Fluo Calibration curve of NATOG prepared in water

In order to assess the levels of NATOG in human urine, a UPLC-MS/MS method was setup. Calibration curves were prepared both in Milli-Q water and two different urine batches (Fig. 1a). All curves showed excellent linear regression, with *R*^*2*^-values > 0.999. Whereas the blank sample prepared in milli-Q water showed no signal at the expected analyte (NATOG) retention time, the blank samples prepared with human urine, showed a signal corresponding to low levels of NATOG (0.31 μM and 0.07 μM), indicating that NATOG is present also in urine from healthy individuals, but at low levels. An example chromatogram of the urine sample with endogenous NATOG concentration of 0.31 μM is presented in Fig. 1b. Quality Control (QC) samples were prepared by spiking NATOG in urine and milli-Q water at 3 different levels: 2 μM (QC-Low), 10 μM (QC-Medium) and 50 μM (QC-High). These QC samples were analyzed in 8-fold in order to assess accuracy and precision of the assay (Table 2). Both accuracy and precision were excellent with accuracy ranging between 96.9 and 112 % and coefficient of variation (CV) lower than 5 % for all samples tested.

**Table 2:** Accuracy and precision of the detection of NATOG using UPLC-MS/MS (*n* = 8)

	QC-Low (2 μM)	QC-Medium (10 μM)	QC-High (50 μM)
Milli-Q Water			
Mean	2.12	10.8	48.5
Standard deviation	0.07	0.40	0.39
Coefficient of variation (%)	3.5	3.7	0.8
% Accuracy	106	108	96.9
Human urine			
Mean	2.23	11.0	55.1
Standard deviation	0.07	0.35	2.32
Coefficient of variation (%)	3.3	3.2	4.2
% Accuracy^a^	112	110	110

^a^Endogenous level of NATOG used for preparation of QC samples was 0.31 μM; results have not been corrected for the endogenous levels

### Evaluation of NATOG stability and adsorption characteristics

Since samples collected in the field might experience exposure to heat and extensive sunlight, a stability study was performed to assess the stability of NATOG under different storage conditions. Results clearly indicate that NATOG is stable for at least 24 h at 50 °C (under different pH conditions) and when exposed to sunlight (3 h in suntestester, corresponding to approximately 36 h of sunlight) (Table 3). As samples are stored in glass or polypropylene (PP) containers, it was investigated whether NATOG adsorbs to these materials. The data presented in Table 3 indicate that this is not the case. It should be noted that the urine sample used for storage stability and adsorption tests had a background NATOG concentration of 5.13 μM.

**Table 3:** Stability and adsorption characteristics of NATOG. Concentrations were obtained after spiking. Samples were analyzed in duplicate. Accuracy of 80–120 % on the theoretical spiked concentration was considered acceptable

	NATOG μM			
Stability^a^	Ref.	24 h 4 °C	24 h RT	24 h 50 °C
Urine pH 4	11.0 ± 0.3	10.7 ± 0.0	11.0 ± 0.4	10.9 ± 0.0
Urine pH 6	10.9 ± 0.3	11.2 ± 0.1	11.4 ± 0.0	11.7 ± 0.1
Urine pH 8	11.6 ± 0.3	11.6 ± 0.1	11.6 ± 0.2	11.8 ± 0.1
Sun stability^b^	Ref.	0.5 h Suntest	3 h Suntest	
Clear vials	2.65 ± 0.01	2.61 ± 0.03	2.57 ± 0.01	
Amber vials	2.64 ± 0.01	2.65 ± 0.01	2.61 ± 0.03	
Adsorption test^a^	Ref. glass	6 cycles glass	Ref. PP	6 cycles PP
Urine pH 4	10.5 ± 0.3	11.0 ± 0.1	10.7 ± 0.1	10.8 ± 0.1
Urine pH 6	11.2 ± 0.1	11.1 ± 0.1	10.7 ± 0.8	11.1 ± 0.1
Urine pH 8	11.3 ± 0.1	11.4 ± 0.1	11.5 ± 0.1	11.3 ± 0.0

^a^Urine with an endogenous NATOG concentration of 5.13 μM was spiked with 5.63 μM NATOG

^b^Sample of 2.81 μM NATOG in Milli-Q water

*Abbreviations*: *Ref.* reference, *RT* room temperature, *PP* Polypropylene

### Performance characteristics of LC-Fluorescence assay for NATOG quantification in urine

Although the simple chromatographic separation used in the LC-MS/MS assay has the potential to be translated into a point-of-care microfluidic method, the use of mass spectrometry for detection would limit its applicability in a field setting. We therefore investigated the possibility of using UPLC followed by fluorescence detection. Chromatographic conditions were taken from the established LC-MS/MS method with slight modification in the chromatographic gradient (see Methods). Detection was performed on a Waters 474 fluorescent detector with excitation and emission wavelengths at 232 nm and 290 nm, respectively. No quenching was observed at 232/290, whereas for 270/290, quenching of the NATOG signal was observed at 2 μM (results not shown). Hence, 232/290 was selected for further sample analysis. Calibration curves were prepared in Milli-Q water and QC samples were prepared in Milli-Q water and urine. Calibration curve showed a nice linear regression (Fig. 1c). Analysis of the QC samples at the different concentrations demonstrated that any interfering compounds present in urine have been separated by the applied chromatography, as comparable concentrations were obtained both in water and urine (Table 4). However, it must be noted that at lower levels (2 μM and 10 μM) slightly higher concentrations were found in urine compared to water, which is caused by an endogenous NATOG level of 0.31 μM in the urine sample used for preparation of QC samples, as determined before in the LC-MS experiments.

**Table 4:** Accuracy and precision of the detection of NATOG using UPLC-Fluorescence (*n* = 8)

	QC-Low (2 μM)	QC-Medium (10 μM)	QC-High (50 μM)
Milli-Q Water			
Mean	2.03	10.2	52.3
Standard deviation	0.02	0.34	1.94
Coefficient of variation (%)	1.0	3.3	3.7
% Accuracy	102	102	105
Human urine			
Mean	2.31	10.3	52.2
Standard deviation	0.08	0.17	0.56
Coefficient of variation (%)	3.5	1.7	1.1
% Accuracy^a^	115	103	104

^a^Endogenous level of NATOG used for preparation of QC samples was 0.31 μM; results have not been corrected for the endogenous levels

**Fig. 2:**
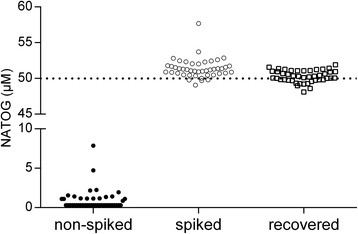
NATOG levels in urine from healthy volunteers from Belgium, both non-spiked and spiked with 50 μM NATOG. NATOG levels were analyzed with the LC-Fluorescence assay. Recovered NATOG is calculated by subtracting the endogenous level (non-spiked) from the total (spiked) concentration

In order to evaluate the NATOG levels in healthy subjects using the LC-Fluorescence assay, a set of 44 urine samples collected from normal healthy volunteers was analyzed (Fig. 2). The majority of subjects had urinary NATOG levels below the quantification limit of 1 μM NATOG and only 36 % of subjects had quantifiable NATOG levels. All but one subject had levels below 5 μM, while one sample had a NATOG concentration of 7.9 μM. As the fluorescent detection of NATOG can be impacted by co-eluting quenchers, all samples were also analyzed after spiking at a final concentration of 50 μM NATOG. After correction for the endogenous NATOG levels, all samples were found to contain NATOG levels of 48–52 μM NATOG, indicating low variability and excellent accuracy using fluorescence detection.

### Assessment of NATOG levels in urine from nodule-positive subjects and controls

**Fig. 3:**
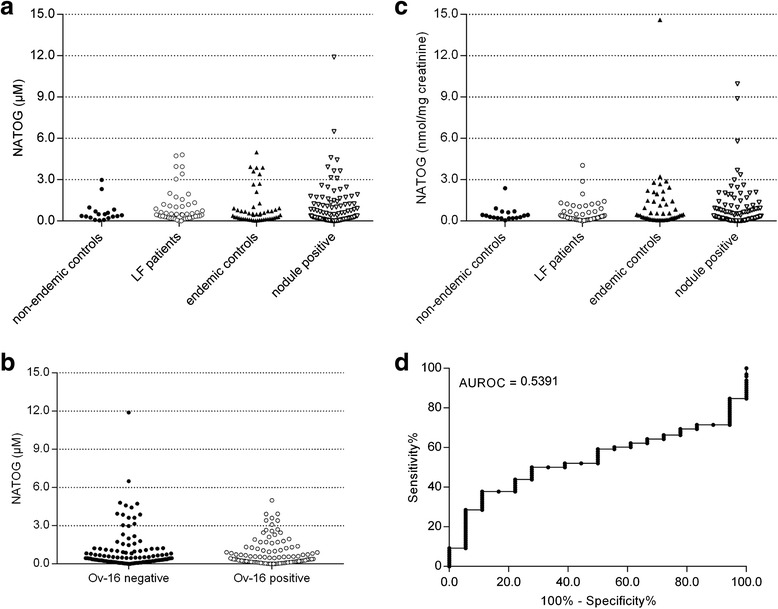
**a** Urinary NATOG levels in urine from nodule-positive individuals in Ghana, endemic controls, non-endemic controls and lymphatic filariasis patients. **b** Comparison of urinary NATOG levels between Ov-16-positive and Ov-16-negative individuals. **c** Normalized NATOG levels using urinary creatinine for normalization. **d** ROC analysis based on the normalized NATOG levels in nodule-positive individuals and non-endemic controls

In order to assess the potential of NATOG as biomarker for diagnosis of *O. volvulus* infection or treatment monitoring, we analyzed, using LC-MS/MS, a series of urine samples from *O. volvulus* infected subjects and endemic controls. Two additional sample sets were included to evaluate the NATOG levels in non-endemic controls and lymphatic filariasis patients. The obtained data are provided in Additional file 1 and summarized in Fig. 3a. Most of the *O. volvulus* infected subjects had very low levels of NATOG in their urine, with an average value of 1.06 ± 0.16 μM (± SEM). Only two infected subjects were identified with NATOG > 5 μM. Furthermore, there was no significant difference between the infected group and endemic controls (0.95 ± 0.18 μM) (Mann–Whitney *U* test: *P* = 0.43). Also lymphatic filariasis patients and non-endemic controls had similar NATOG levels with an average value of 0.99 ± 0.17 μM and 0.66 ± 0.18 μM, respectively. Further examination showed there was no significant difference between samples from Ov-16-positive individuals and Ov-16-negative individuals either (Mann–Whitney *U* test: *P* = 0.23, Fig. 3b).

Since urine volume can highly vary upon water consumption, environmental factors and physiological or pathological factors, metabolite concentrations in urine also may vary widely [32]. Biomarker concentrations are therefore commonly expressed as a ratio to the urinary creatinine concentration. We therefore measured creatinine levels in all urine samples and normalized the NATOG results based on the corresponding creatinine value. These normalized data are presented in Fig. 3c. Although some differences can be observed compared to the non-normalized data, there still is no statistical difference between any of the groups investigated. ROC analysis on the normalized data of nodule-positive and non-endemic controls (the groups that differ the most), also indicate that urinary NATOG is not a powerful biomarker for discriminating both groups, even after normalization (AUROC = 0.54, Fig. 3d).

## Discussion

Previous work by Globisch et al. [25] found *N*-acetyltyramine-*O*,β-glucuronide (NATOG) in urine as a unique biomarker for *O. volvulus* infection. We have further investigated the diagnostic utility of this biomarker and evaluated its use in field conditions in predominantly rural settings in sub-Saharan Africa. Essential for this is the stability of the metabolite at elevated temperatures and upon extensive sunlight exposure. We found that NATOG has well-acceptable stability characteristics with stability in urine being demonstrated upon incubation at 50 °C for 24 h and exposure to extensive light equivalent to 36 h sunlight. Also no adsorption was observed to glass or polypropylene tubes. These characteristics would ease collection of samples from remote areas and transportation to a central lab for analysis of urinary NATOG. However, although centralized analyses are possible, not many local laboratories have access to LC-MS/MS capabilities. We have therefore developed an LC-Fluorescence method for quantification of NATOG in urine. On the one hand, this technology is more readily available in those regions, but on the other hand it also opens the path for development of microfluidic tools that could then be used for point-of-care assessment of NATOG. We have shown here that fluorescent detection preceded by simple chromatographic separation is feasible and does not require any extraction procedure. This offers the possibility to convert this LC-Fluorescence assay into a simple test consisting of a microfluidic chromatography system coupled to fluorescence detector using a laser or light emitting diode (LED) as the excitation light source [33, 34].

In the last part of our investigation, we evaluated the diagnostic accuracy of urinary NATOG as biomarker for *O. volvulus* infection. In this sample set, collected from Ghana, we could not detect significantly elevated levels (> 20 μM) in urine from infected subjects. Most samples had NATOG levels below 5 μM. Only two samples out of 98 appeared to have slightly elevated levels of NATOG with 12 μM being the highest level observed, which was still far from the previously observed concentrations that reached levels above 100 μM in some individuals. Also, when compared to the levels in urine from non-infected controls (both endemic and non-endemic), no statistical difference could be observed since also in these subjects urinary NATOG levels up till 5 μM were found. Furthermore, also in one of the urine batches that were used for preparation of the stability samples and that were donated by healthy volunteers from Belgium, 5 μM NATOG was detected. All these observations together can be summarized in two main conclusions: (i) non-infected individuals usually have very low levels of urinary NATOG, but under certain circumstances, these levels might slightly increase; and (ii) in nodule-positive subjects from Ghana no clearly elevated levels of urinary NATOG can be detected. Since urine can be more or less diluted, we also examined the data after normalization with urinary creatinine. It is assumed that urinary creatinine excretion rate is constant within an individual over time and across individuals, and that biomarker production or excretion has a linear relationship with urinary creatinine excretion across individuals [35]. Normalization of the data however did not affect any of the conclusions.

One explanation for the discrepancy between these and previous observations might be the fact that in this study all nodule-positive subjects are from a community that participates in a mass drug administration program and maybe more importantly, all subjects had no or very low levels of microfilaria detected in the skin. It is likely that an active infection, with clear microfilaridermic features, is required for urinary NATOG to increase to a level that differs significantly from the levels in uninfected individuals. This would still value NATOG as a biomarker for *O. volvulus* infection, but more specifically to identify microfilaridermic patients with active infection and to be able to monitor treatment efficacy in them. In this respect, it should be noted that in previous work also samples were included from infected patients who were treated with ivermectin or ivermectin and doxycycline [25]. Remarkably, ivermectin treatment alone did not result in a reduction of urinary NATOG levels while treatment with doxycycline (and ivermectin) did cause a dramatic drop in the NATOG levels in urine samples, collected 20 months after treatment. Whereas ivermectin has direct microfilaricidal activity, doxycycline kills the endosymbiotic bacteria *Wolbachia*, thereby sterilizing the adult female worm, ultimately also resulting in a reduced microfilarial load [36–40]. Detailed analysis of the samples that were used in this assessment however, demonstrated that in the group treated only with ivermectin, a substantial amount of microfilaria are still present in the skin while no or very low numbers were detected in the group treated with doxycycline and ivermectin [41]. This observation supports the hypothesis that urinary NATOG is indeed elevated in case of active infection with substantial levels of microfilaria in the skin. This hypothesis is further supported by the fact that the samples used in this study were collected along the Pra River, in an area that has been shown to have no active onchocerciasis transmission. This is caused by the ceased blackfly breading due to the high water turbidities in the river, caused by the intensification of the gold-digging activities along the river [42]. This would imply that all nodule-positive individuals have older adult worms (>3 years) which are shown to be morphologically and immunohistologically different from younger worms [43]. These older worms often are found to be senescent with empty uteri, signs of degeneration and in some cases already might have died, also as a result of consecutive rounds of ivermectin treatment [43, 44]. It is very likely to assume that these older worms produce much lower levels of tyramine compared to their younger and more active counterparts.

An alternative explanation for the different observations is the possibility that NATOG is not, or not only produced as a result of *O. volvulus* infection but is in fact the result of the metabolism of tyramine absorbed from the gut. Although Globisch and coworkers indicated that tyramine acetylation, the first step in the metabolism of tyramine to NATOG, is specifically performed by *O. volvulus* enzymes before being secreted into the host for further metabolism and excretion, it is well possible that this acetylation step is performed by a host liver enzyme or by enzymes in the gastrointestinal mucosa [25, 45, 46]. Consequently, increased levels of tyramine in the gut, either directly from food or after production by gut bacteria, may result in increased urinary NATOG levels. In this respect, it is well-known that tyramine is adsorbed in the body upon consumption of e.g. fermented cheese and wine, with *Enterococcus faecalis* being one of the most important bacteria producing tyramine in fermented food products [47–51]. It is however not clear whether this could lead to the very high levels (> 100 μM) that were observed in *O. volvulus-*infected individuals [25]. Further research will be required to better characterize the origin of NATOG in urine and to elucidate the role the gut flora might play.

## Conclusions

Our data show that urinary NATOG as a diagnostic biomarker for *O. volvulus* infection appears not to be useful in patients with very low levels of microfilaria in the skin and indicate that NATOG most likely can only be used as biomarker of a highly productive infection of *O. volvulus*. More studies where urinary NATOG is measured in patients with different levels or stages of infection will be required to confirm these conclusions.

## Additional file

Additional file 1: Overview of data obtained for all samples (urinary NATOG, urinary creatinine and Ov16 status). (XLSX 18 kb)

## Abbreviations

NATOG: *N*-acetyltyramine-*O*,β-glucuronide
Mf: microfilaria
LC-MS/MS: liquid chromatography tandem mass spectrometry
UPLC: Ultra Performance Liquid Chromatography
QC: Quality Control
